# Fast Food Consumption and Gestational Diabetes Incidence in the SUN Project

**DOI:** 10.1371/journal.pone.0106627

**Published:** 2014-09-12

**Authors:** Ligia J. Dominguez, Miguel A. Martínez-González, Francisco Javier Basterra-Gortari, Alfredo Gea, Mario Barbagallo, Maira Bes-Rastrollo

**Affiliations:** 1 Geriatric Unit - Department of Internal Medicine and Specialties, University of Palermo, Palermo, Sicily, Italy; 2 Department of Preventive Medicine and Public Health, University of Navarra, Pamplona, Navarra, Spain, and CIBER Fisiopatologia de la Obesidad y Nutricion (CIBERobn), Instituto de Salud Carlos III, Madrid, Spain; 3 Department of Internal Medicine (Endocrinology), Hospital Reina Sofia, Tudela, Navarra, Spain; Medical University Innsbruck, Austria

## Abstract

**Background:**

Gestational diabetes prevalence is increasing, mostly because obesity among women of reproductive age is continuously escalating. We aimed to investigate the incidence of gestational diabetes according to the consumption of fast food in a cohort of university graduates.

**Methods:**

The prospective dynamic “Seguimiento Universidad de Navarra” (SUN) cohort included data of 3,048 women initially free of diabetes or previous gestational diabetes who reported at least one pregnancy between December 1999 and March 2011. Fast food consumption was assessed through a validated 136-item semi-quantitative food frequency questionnaire. Fast food was defined as the consumption of hamburgers, sausages, and pizza. Three categories of fast food were established: low (0–3 servings/month), intermediate (>3 servings/month and ≤2 servings/week) and high (>2 servings/week). Non-conditional logistic regression models were used to adjust for potential confounders.

**Results:**

We identified 159 incident cases of gestational diabetes during follow-up. After adjusting for age, baseline body mass index, total energy intake, smoking, physical activity, family history of diabetes, cardiovascular disease/hypertension at baseline, parity, adherence to Mediterranean dietary pattern, alcohol intake, fiber intake, and sugar-sweetened soft drinks consumption, fast food consumption was significantly associated with a higher risk of incident gestational diabetes, with multivariate adjusted OR of 1.31 (95% conficence interval [CI]:0.81–2.13) and 1.86 (95% CI: 1.13–3.06) for the intermediate and high categories, respectively, versus the lowest category of baseline fast food consumption (p for linear trend: 0.007).

**Conclusion:**

Our results suggest that pre-pregnancy higher consumption of fast food is an independent risk factor for gestational diabetes.

## Introduction

Gestational diabetes mellitus (GDM), traditionally defined as carbohydrate intolerance first diagnosed during pregnancy [1], has long been recognized as a risk factor for a number of unfavorable outcomes. These include short- and long-term complications for mothers (i.e., preeclampsia or eclampsia, and type 2 diabetes after delivery), and for offspring (i.e., macrosomia, increased likelihood of trauma at birth, cesarean delivery, and neonatal metabolic abnormalities, such as hypoglycemia or hyperbilirubinemia) [1], [2]. GDM prevalence is increasing, mostly because obesity among women of reproductive age is continuously escalating [3]–[5]. It has been reported that gestational diabetes affects 1–14% of all pregnancies in the US [1], and about 2–6%of pregnancies in Europe [5]. A recent meta-analysis of RCTs suggested that interventions on glucose control/monitoring, diet, or pharmacological treatment including insulin, did not significantly reduce the risk of adverse outcomes (i.e. cesarean delivery and perinatal or neonatal death) [6]. Hence, the identification of modifiable risk factors, such as excess adiposity, decreased physical activity, and unhealthy diet for the prevention of gestational diabetes is criticalin order to avoid associated harmful outcomes [7].

Several observational studies have related some pre-pregnancy dietary factors with GDM risk. An inverse association between pre-pregnancy adherence to healthful dietary patterns and gestational diabetes incidence has been reported [8]. Low fiber intakes or high dietary glycemic index [9], high red meat/processed meat consumptions [10], and high intakes of animal fat and cholesterol [11] have all been associated with an elevated risk of GDM.

Previous studies have suggested that Western-style fast food, which is calorically dense and usually served in large portions, is a determinant of weight gain, insulin resistance, and type 2 diabetes incidence [12], [13]. However, the association of fast food consumption with GDM risk remains unknown. Therefore, we conducted the present analyses to appraise whether fast food consumption (hamburgers, sausages, and pizza) was associated with GDM risk in the SUN project (“Seguimiento Universidad de Navarra”, University of Navarra Follow-up).

## Materials and Methods

### Study population

The SUN project is a prospective dynamic cohort study entirely composed of university graduates. The recruitment started in 1999 and it is permanently open. The design and methods utilized in the SUN study have been previously described in detail [14], [15]. In brief, graduates from the University of Navarra and other Spanish universities, registered nurses and other health professionals from different Spanish provinces were invited to participate by a mailed questionnaire. The study protocol was approved by the Institutional Review Board of the University of Navarra and the initial response to the questionnaire was considered as informed consent to participate. After the baseline assessment, participants receive a follow-up questionnaire every two years on diet, lifestyle, risk factors, and medical conditions.

**Figure 1:**
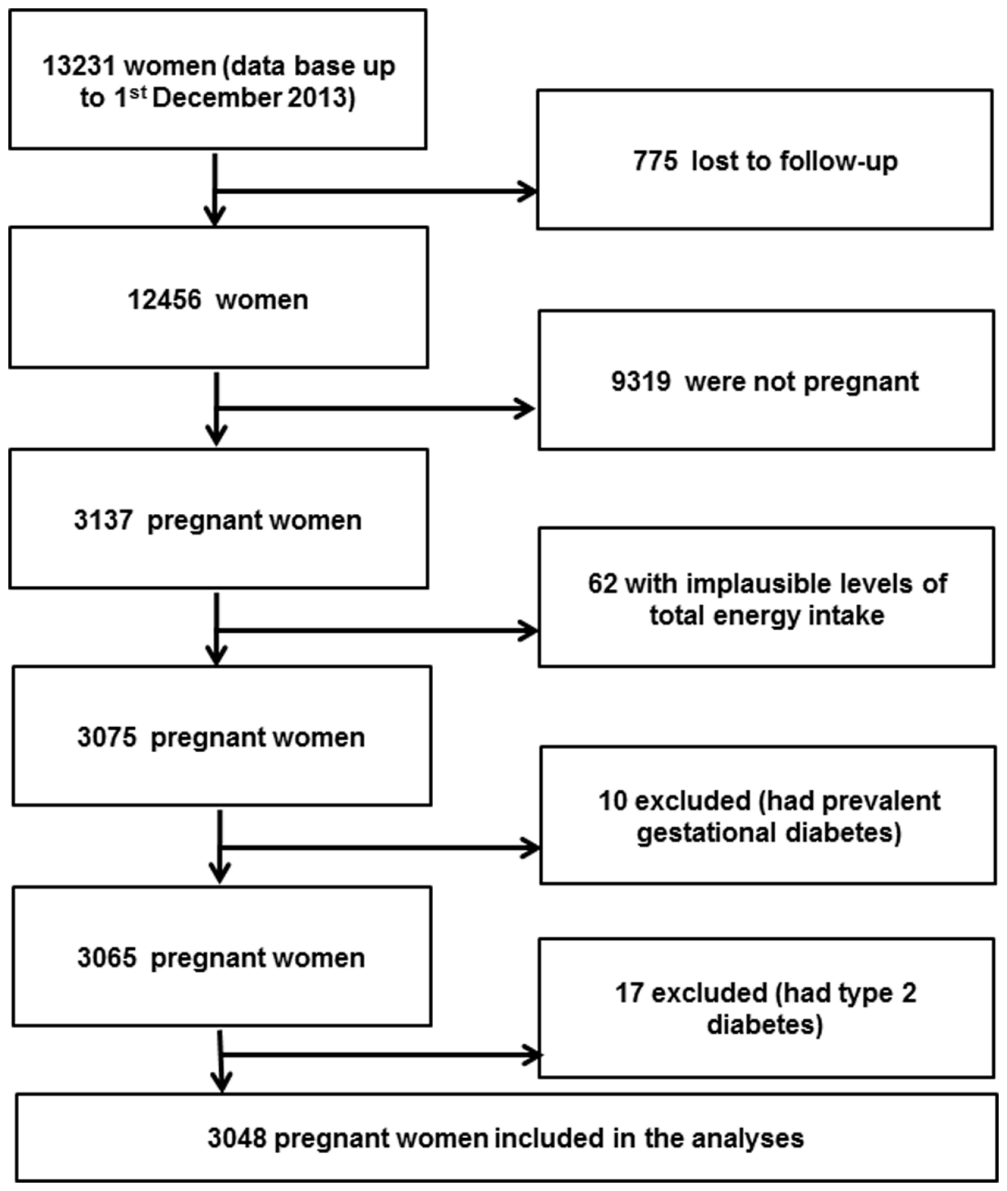
Flow chart depicting the selection process among participants of the SUN project to be included in the present analyses.

For the present analyses we examined the last available database as of the 1^st^ of December 2013. From a total 13,231 women, we included 12,456 women who had answered the baseline questionnaire before the 1^st^ of March 2011, to have enough time to answer the first follow-up questionnaire. Up to that date, 3,137 pregnant women were identified among them. Women were excluded from the analyses if they reported extremely low (below percentile 1) or high (above percentile 99) values for total energy intake (n = 67), had prevalent or previous gestational diabetes (n = 10), or had a previous diagnosis of diabetes (n = 17). Women who reported gestational diabetes in a previous pregnancy were not included in the analyses because they were thought to be more likely to have changed their diet and lifestyle during the next pregnancy to prevent recurrent gestational diabetes. The final analytic population included 3,048 pregnant women (**Fig. 1**).

Since we have excluded those pregnant women with implausible levels of total energy intake, missing data on fast food consumption (n = 40) was considered as no consumption. Those with missing values in smoking were treated as another category (current, former smoker, never smokers, and missing [n = 67]). For missing anthropometrics data we used the last value carried forward, although in this sample of the SUN Project there were no missing data for these variables.

### Dietary assessment

Dietary habits at baseline were assessed by a semi-quantitative food frequency questionnaire (FFQ) with 136 items previously validated and described in detail [16], [17]. The validity [18] and reproducibility [19] of this questionnaire have been also recently assessed. Updated Spanish food composition tables were used to assess nutrient intake. Nutrient scores were computed using an *ad hoc* computer software specifically developed for this aim. A trained dietitian updated the nutrient data bank using the latest available information included in food composition tables for Spain [20], [21]. Adherence to the Mediterranean food pattern was appraised using the sample-specific score proposed by Trichopoulou [22]. Fast food was defined as the consumption of hamburgers, sausages, and pizza, as previously reported [23], [24]. We estimated energy adjusted fast food consumption through the residuals method [25]. Three categories of fast food (serving  = 100 g) consumption were also established: low (0–3 servings/month), intermediate (>3 servings/month and ≤2 servings/week) and high (>2 servings/week).

### Ascertainment of gestational diabetes

The outcome of interest was the incidence of gestational diabetes. Pregnant women identified in the SUN project (**Fig. 1**) who reported a diagnosis of GDM made by a physician in the biennial questionnaire and did not have diabetes at baseline were considered as incident cases of new-onset GDM.

We sent an additional questionnaire to participants who reported probable new onset GDM, requesting their written confirmation and date of the diagnosis of either GDM, a previous diagnosis of diabetes, their highest fasting glucose value, their first glycated haemoglobin during pregnancy, whether they had ever undergone an oral glucose tolerance test and its results, and the use during pregnancy of insulin. We also asked them to send us the medical report detailing their diagnoses. A panel of medical doctors, blinded to the information about dietary habits, used the information provided by participants (additional questions and medical reports) to classify the diagnosis or each candidate woman as an incident case of GDM or not. For the present analyses we considered only those cases of confirmed GDM. From the potential cases of GDM we had avalaible information in 98% of them. Among those with information, 80% of them were confirmed as incident cases of GDM. There is a lack of universally accepted diagnostic criteria for GDM [5]. Several different protocols are in regular use internationally, each with its own recommendations on which pregnant women should be selected for biochemical testing, how the test should be performed and what glycemic thresholds should be considered diagnostic [26]. The usual diagnosis criteria for GDM in Spain were those with 100-g oral glucose tolerance test with the cut-off points of Carpenter and Coustan [27] or the cut-off points from the National Diabetes Data Group [28] after a positive 50-g glucose challenge test.

### Other covariates

Information on other covariates was assessed in the baseline questionnaire. This included socio-demographic parameters (age), anthropometric measurements (weight, body mass index [BMI]), health related habits (smoking status, physical activity, sedentary lifestyle), and clinical variables (use of medication, self-reported pregnancy, family history of diabetes, cardiovascular disease/hypertension, parity). Age was calculated from the date of birth to the date of the questionnaire's return. Self-reported weight and BMI have previously shown a high validity in a specific study conducted in a sub-sample of this cohort [29 27]. Physical activity was assessed using a previously validated questionnaire with a Spearman correlation coefficient of 0.51 (p<0.001) between questionnaire information and objectively obtained measurements [30]. Physical activity was expressed in metabolic equivalent tasks (METs/weeks) as calculated from the time spent at each activity in hours per week multiplied by its typical energy expenditure [31].

### Statistical analysis

Consumption frequencies of the fast food items considered (hamburgers, sausages, and pizza) were standardized, summed, and divided into categories that allowed logical cut points with a sufficient number of subjects. Means with SDs for continuous baseline characteristics and proportions for categorical characteristics were calculated by categories of fast food frequency consumption. Since the risk of gestational diabetes depends on the fact to be pregnant and not on a timely basis, we estimated the odds ratios (and 95% CIs) for each of the three categories of fast food consumption using non-conditional regression models taking as the reference category those women with the lowest fast food consumption (0–3 servings per month). For each exposure category, we fitted a crude (univariate) model, an age-adjusted model (Model 1), and multivariate models with additional adjustments (Models 2 and 3) for a priori-selected pre-pregnancy dietary and non-dietary covariables (see below). Non-conditional logistic regression models were adjusted for age, BMI (Kg/m^2^), total energy intake (Kcal/day), smoking status (never, former and current smokers), physical activity (expressed in METs-h/w), family history of diabetes (yes or no), presence at baseline of cardiovascular disease or hypertension (yes or no), parity (first pregnancy/1 or 2 pregnancies before/3 or more pregnancies before), score of adherence to the Mediterranean dietary pattern (0–9 points score), and other potential dietary confounders, such as fiber intake (g/day), and sugar-sweetened soft drink consumption (ml/day). The p for trend was calculated taking the median consumption of fast food for each category and introducing this new variable as a continuous variable in the models. We evaluated the interaction between fast food consumption and sugar-sweetened soft drink consumption, physical activity, and family history of diabetes on the risk of GDM through likelihood ratio tests for each of the product-terms introduced (each at one time) in fully-adjusted models.

The analyses were performed with Stata software package version 12 (Stata Corp). All tests were two sided and statistical significance was set at the conventional cut-off of p<0.05.

## Results

Baseline characteristics of the studied population of pregnant women in the SUN project, according to categories of fast food consumption (low to high), are shown in **Table 1** **.** Almost half of the participants had an intermediate consumption of fast food (>3 servings/month and ≤2 servings/week; 48%) whereas lower values (0–3 servings/week; 20%) were less frequently observed. Higher frequency of fast food consumption (>2 servings/week; 32%) was present in nearly a third of participants. The participants with a higher consumption of fast food were on average younger, more likely to be current smokers, multiparous, less physically active, less adherent to the Mediterranean dietary pattern, and also more likely to have a lower fiber intake and a higher fat intake.

**Table 1:** Characteristics of 3,048 pregnant women in the SUN cohort according to their frequency of fast food consumption.

	Fastfood consumption		
	0–3 servings*/month	>3 servings/month and ≤2 servings/week	>2 servings/week
N (%)	616 (20.2)	1461 (47.9)	971 (31.9)
Age (years)	29.3 (5.5)	28.9 (4.6)	28.6 (4.4)
Gestational diabetes (%)	3.9	4.8	6.7
Family history of diabetes (%)	10.4	11.2	9.8
Current smoking (%)	22.2	25.0	26.6
Body Mass Index (kg/m^2^)	21.3 (2.6)	21.4 (2.6)	21.6 (2.7)
Nulliparous (%)	81.0	81.1	77.9
Physical activity (METs-h/week)	22.4 (23.8)	17.9 (18.2)	16.7 (19.1)
Prevalence of hypertension (%)	2.6	2.4	1.7
Prevalence of CVD (%)	0.7	0.7	0.5
Mediterranean diet score	4.6 (1.7)	4.5 (1.7)	3.9 (1.7)
Alcohol intake (g/d)	3.3 (4.9)	4.0 (5.2)	3.6 (4.7)
Fiber intake (g/d)	32.9 (17.0)	28.2 (11.7)	25.4 (10.9)
Total energy intake (kcal/d)	2479 (842)	2357 (685)	2395 (689)
Carbohydrate intake (% energy)	43.6 (8.8)	42.5 (7.2)	41.4 (6.7)
Protein intake (% energy)	18.7 (3.7)	18.6 (3.0)	18.4 (2.9)
Fat intake (% energy)	36.7 (7.9)	37.6 (6.2)	39.1 (6.1)
SFA intake (% energy)	12.0 (4.0)	12.8 (3.1)	13.5 (2.9)
MUFA intake (% energy)	16.0 (4.5)	16.2 (3.8)	16.4 (3.4)
PUFA intake (% energy)	5.4 (1.9)	5.4 (1.6)	5.7 (1.7)
Trans fatty acid intake (% energy)	0.3 (0.2)	0.4 (0.2)	0.4 (0.2)
Soft drinks (servings/week)	1.3 (2.9)	1.5 (2.4)	1.9 (2.9)

*1 serving = 100 g.

*METs: metabolic equivalent tasks; CVD: cardiovascular disease; SFA: saturated fatty acid; MUFA: monounsaturated fatty acid; PUFA: polyunsaturated fatty acid*.

Values are means (SD) for age, BMI, physical activity, Mediterranean diet score, alcohol, fiber, and total energy intake.

**Figure 2:**
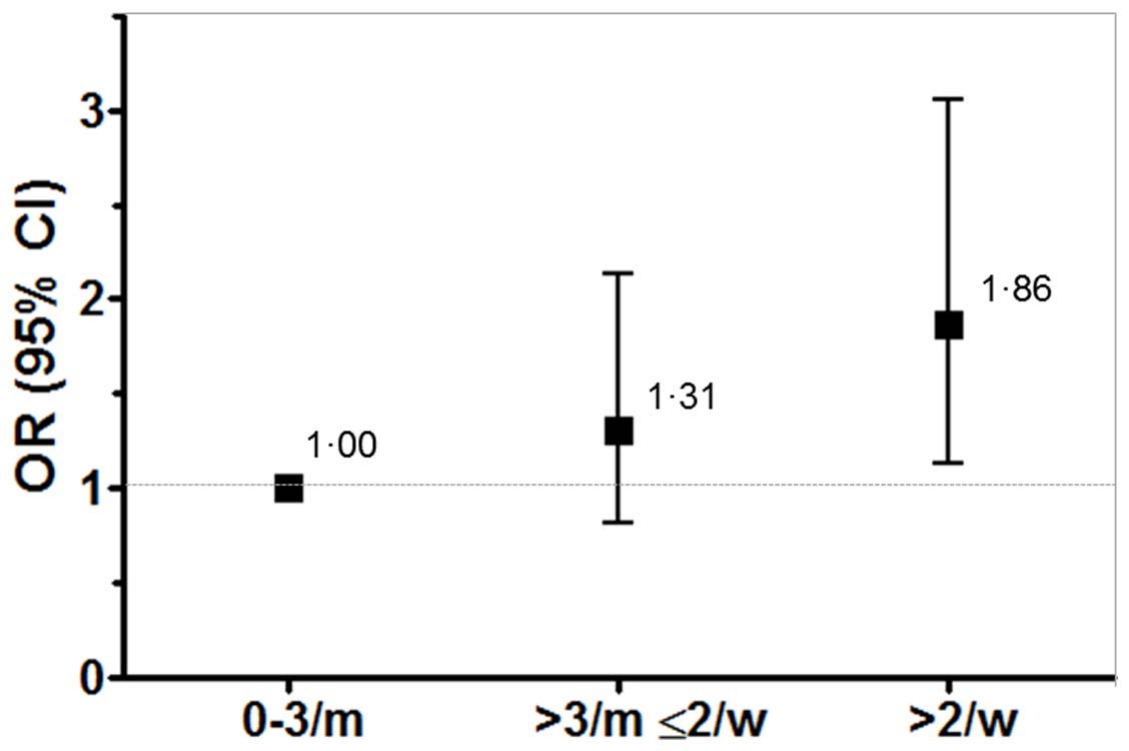
Odds ratio (OR) and 95% confidence interval (CI) for incident gestational diabetes according to frequency of fast food consumption in the SUN project (n = 3,048 pregnant women). Respective numbers (gestational diabetes incidence) for fast food intake of 0–3 times per month (low), >3 times a month and ≤2 times per week (intermediate), and >2 times per week (high) were 616 (24), 1,461 (70), 971 (65). Results represent fully adjusted model (age, baseline BMI, total energy intake, smoking, physical activity, family history of diabetes, cardiovascular disease/hypertension at baseline, parity, adherence to Mediterranean dietary pattern, fiber intake, alcohol intake, and sugar-sweetened soft drinks consumption).

During 28,064 person-year follow-up (mean follow-up: 10.2, sd: 2.9 years) 159 women reported a first diagnosis of GDM among 3,048 pregnant women of the SUN project, corresponding to 5.2% of pregnant participants. When we analyzed the association between the consumption of fast food and incident gestational diabetes during follow-up with adjustments for a set of potential dietary and non-dietary confounders (age, baseline BMI, total energy intake, smoking, physical activity, family history of diabetes, cardiovascular disease/hypertension at baseline, parity, adherence to Mediterranean dietary pattern score, fiber intake, and sugar-sweetened soft drinks consumption), we found that fast food consumption was strongly and positively associated with incident gestational diabetes risk. In fully-adjusted models, those women in the highest category of fast food consumption presented almost a twofold risk of developing GDM (adjusted OR: 1.86; 95% CI: 1.13–3.06) compared to pregnant women with the lowest fast food consumption (0–3 servings per month) (**Table 2** **and** **Fig. 2**).The risk of incident gestational diabetes was 3.9% for those in the lowest category of fast food consumption, 4.8% for those with intermediate fast food consumption, and 6.7% for the highest category. These associations remained statistically significant after further adjustment for the presence of polycystic ovary syndrome (**Table 2**).

**Table 2:** OR and 95% confidence interval of incident gestational diabetes according to fast food consumption.

	Fastfood consumption			
	0–3 servings*/month	>3 servings/month and ≤2 servings/week	>2 servings/week	p for trend
Cases, n/N	24/616	70/1461	65/971	
Rate	4.11*10^−3^	5.16*10^−3^	7.41*10^−3^	
Model 1: OR (95% CI)	1.0 (Ref.)	1.26 (0.78–2.02)	1.80 (1.11–2.91)	0.009
Model 2: OR (95% CI)	1.0 (Ref.)	1.31 (0.81–2.12)	1.90 (1.15–3.12)	0.005
Model 3: OR (95% CI)	1.0 (Ref.)	1.31 (0.81–2.13)	1.86 (1.13–3.06)	0.007
Model 2: OR (95% CI) and PCO	1.0 (Ref.)	1.31 (0.81–2.13)	1.90 (1.16–3.13)	0.005
Model 3: OR (95% CI) and PCO	1.0 (Ref.)	1.31 (0.81–2.13)	1.86 (1.13–3.07)	0.007
Model 2: OR (95% CI) w/o multiparous	1.0 (Ref.)	1.07 (0.64–1.79)	1.71 (1.01–2.90)	0.013
Model 3: OR (95% CI) w/o multiparous	1.0 (Ref.)	1.07 (0.64–1.79)	1.70 (1.00–2.89)	0.014
Model 2: OR (95% CI) w/o obese	1.0 (Ref.)	1.25 (0.77–2.04)	1.83 (1.11–3.03)	0.008
Model 3: OR (95% CI) w/o obese	1.0 (Ref.)	1.25 (0.77–2.04)	1.81 (1.10–2.99)	0.010

The SUN project 1999–2012.

*1 serving = 100 g.

*OR: odd ratio; CI: confidence interval; Rate: crude incident gestational diabetes rate per 10,000 person-years; PCO: polycystic ovary syndrome; w/o: without*.

*Model 1: adjusted for age*.

*Model 2: model 1 plus adjustment for total energy intake, smoking, physical activity, family history of diabetes, cardiovascular disease/hypertension at baseline, parity, adherence to Mediterranean dietary pattern score, alcohol intake, fiber intake, and sugar-sweetened soft drinks consumption*.

*Model 3: model 2 plus adjustment for baseline BMI*.

When we used tertiles of energy-adjusted fast food consumption the results were similar: we observed an adjusted OR of 1.82 (95% CI 1.18–2.78) for the highest versus the lowest tertile (p for trend: 0.004).

No significant interaction was observed between fast food consumption and sugar-sweetened soft drinks consumption (p for interaction: 0.645) or between fast food consumption and physical activity (p for interaction: 0.280).

When we assessed fast food consumption as a continuous variable, each additional serving (100 g) per day of fast food consumption was associated with an increased risk, although this association was not statistically significant (adjusted OR: 1.42; 95% CI: 0.76–2.63).

Several sensitivity analyses were carried out in order to appraise the robustness of our findings. This included restricting our analysis to first births from nulliparous women to reduce possible confounding by experiences from previous pregnancies. Excluding multiparous women, the results of the fully adjusted model did not change materially (highest consumption: OR 1.70 (95% CI: 1.00–2.89 for highest vs. lowest consumption; p for linear trend: 0.014). The exclusion of obese women did not either change the results of the fully adjusted model (highest consumption: OR 1.81 (95% CI: 1.10–2.99 vs. low consumption; p for linear trend: 0.010).

To test the existence of a potential selection bias due to lost during follow-up we compared baseline characteristics between those participants included in the analyses and those lost during follow-up. There were no statistical significant differences between both groups.

## Discussion

The results of the present analyses, using data from a large, well-characterized, prospective cohort of Spanish university graduates, found that among pregnant women in the cohort, a higher pre-pregnancy consumption of fast food was associated with a significantly higher risk of developing gestational diabetes. This association remained significant after several adjustments for potential confounders. Our findings are relevant in the context of a global epidemic of diabetes, which is likely driven, at least in part, by an unhealthy Westernized diet and lifestyle [32]. Fast food is a hallmark of such unhealthy diet; hence, the present results help to reinforce its harmful consequences in women of reproductive age.

It is widely accepted that one of the characteristics of normal pregnancy is a state of insulin resistance with a relative intolerance to dietary carbohydrates and a compensatory increase in insulin secretion from the beta cells in the pancreas [1], [33]. The pregnancy-related insulin resistance mainly occurs after the second trimester when insulin requirements are higher. Pregnant women who develop gestational diabetes are assumed to have a compromised beta cell capacity unable to adapt to the increased demand of insulin due to target-organ insulin resistance. This resembles closely the pathophysiology of type 2 diabetes [34], thus, pregnancy-related metabolic challenges may unmask a predisposition to glucose metabolic disorders, which may attain hyperglycemia in the range of diabetic disease. Furthermore, gestational diabetes is a recognized risk factor for future development of type 2 diabetes [35].This and other unfavorable outcomes of gestational diabetes, from delivery complications to perinatal mortality, call for efforts to identify modifiable factors, such as an unhealthy diet, predisposing to the condition.

We found an incidence of gestational diabetes (5.8%) that was similar to that previously reported in the Nurses' Health Study (6.4%) [8]. Attempts to identify women at risk of developing gestational diabetes have been conventionally directed to socio-demographic characteristics, such as maternal weight/BMI, and family history of type 2 diabetes [1], [2]. However, in the past decade, several investigations have been focused on the pregravid dietary and lifestyle factors that may contribute to such risk, in parallel with studies pointing to these factors as main drivers of type 2 diabetes epidemic. Even if former studies showed inconsistentresults on the role of total dietary fat intake and gestational diabetes risk, two recent studies taking in consideration specific types of fat concluded that higher pre-pregnancy consumptions of animal fat and cholesterol [11], and of saturated fat [36] were associated with an elevated risk of gestational diabetes. Likewise, a very recent study reported that a higher intake of animal protein, particularly red meat, was significantly associated with a greater risk of gestational diabetes. Conversely, a greater intake of vegetable protein, in particular nuts, was associated with a significantly lower risk [37]. Our findings are consistent with these studies, since the components of fast food considered in our study (hamburgers, sausages, and pizza) mainly include animal proteins and saturated fat. Our results also showed that pregnant women with a higher consumption of fast food had a lower dietary fiber intake and were less adherent to the Mediterranean dietary pattern. In accordance with our observations, previous study have reported inverse and significant associations for fiber intake [9] and adherence to healthful dietary patterns, including Mediterranean diet [8], and the risk of developing gestational diabetes.

Even if the precise molecular mechanisms are unclear, the observed associations between pre-pregnancy fast food consumption and gestational diabetes risk are biologically plausible. First, maternal pregravid BMI has been linked to gestational diabetes risk [1], [2] and Western-style fast food has been identified among the greatest dietary contributors to weight gain [38]. However, our results were significant even when the data were adjusted for baseline BMI. Second, there is evidence that frequent consumption of Western-style fast food contributes to insulin resistance [12], a mechanism that is central, as mentioned above, in the development of gestational diabetes. Third, saturated fat and cholesterol, which are components of red and processed meats (part of fast food in our study), have shown to adversely affect not only insulin sensitivity but also beta cell function [39], relevant in the pathophysiology of gestational diabetes. Other components of red and processed meats, including heme iron, and nitrosamines may add to oxidative stress and beta cell damage [9].

It has been previously shown that a ‘fast food’ dietary pattern was associated with weight gain rate during pregnancy in a dose-dependent manner in a study conducted in Finland [40]. The result of that study is indirectly supportive of our findings. The independent association between pregravid fast food consumption and GDM incidence that we observed was present even after adjustment for baseline BMI. Indeed, we may wonder whether this association was driven by gestational weight gain among fast food eaters or by other mechanisms. Unfortunately, we do not have specific available information to track weight changes in detail throughout the gravid period of our participants and we cannot provide further specific analyses to assess whether gestational weight changes are the most important mediators of this association. However, overadjustment for intermediate mechanisms through which fast food consumption can increase the risk of GDM would be inappropriate if the aim of our analyses is to control for confounding. It is recognized that in an attempt to estimate the total effect of an exposure on some outcome, control for intermediate factors in the causal chain (that is, overadjustment) will generally bias estimates of the total effect of the exposure on the outcome [41], [42].

The global epidemic of diabetes, including increasing rates of gestational diabetes [43], parallels obesity trends worldwide. These “twin epidemics” have been linked to Westernization of diet and lifestyle [32], [44]. Fast food advertising and broad accessibility may have considerable influence on food choices, particularly among young people. This includes women of childbearing age, increasing their risk for gestational diabetes. In our study, women with the highest consumption of fast food were younger and more likely to have other unhealthy behaviors (i.e. sedentary, smoking, and lower adherence to Mediterranean dietary pattern). Thus, fast food consumption could be a marker of an unhealthy diet/lifestyle and not a cause of increased gestational diabetes risk. However, the multivariate analyses we present here accounted for other unhealthy behaviors, and after multiple adjustments and sensitivity analyses the consumption of fast food remained strongly significantly associated with gestational diabetes risk. Emphasis is needed to develop strategies that persuade women to optimize their diet and lifestyle before conceiving.

The present study has a number of strengths, including a large sample size with a high retention rate, prospective design, a lengthy follow-up and use of a FFQ that has been specifically and repeatedly validated in Spain [17]–[19]. Importantly, we were able to obtain a high degree of control for confounding, including potential lifestyle and demographic confounders, and we also controlled for overall dietary patterns. In addition, we conducted several sensitvity analyses and the results were robust. The study also has some potential limitations. Information that was self-reported and this may lead to some degree of misclassification, which most often would drive associations toward the null value. However, parameters such as self-reported weight and BMI have been previously validated in a sub-sample of this cohort [29]. The cohort is composed mostly of middle-aged, Spanish highly educated persons.Therefore the generalizability of our results must be based on common biological mechanisms instead of on statistical representativeness, and we used restriction to reduce potential confounding by disease, education, socioeconomic status, and presumed access to health care. Future studies are needed in order to test the applicability of our results in pregnant women from other populations.

Since our all participants are Spanish university graduates, we can guarantee that the vast majority of them are Caucasians and therefore, unfortunately we were not able to assess the effect of ethnicity in the association between fast food consumption and GDM.

Finally, possible concerns may arise from the use of dietary data derived from an FFQ, which may be subject to information bias. However, the FFQ used has been repeatedly validated [17]–[19]. Moreover, it is difficult to find a better alternative than a FFQ as an advantageous method to characterize the food habits of large samples of individuals' that need to be followed-up over long periods of time in order to rank them and assess associations with incident clinical end-points [45].

In conclusion, findings from this prospective study suggest that pre-pregnancy higher consumption of fast food (i.e. hamburgers, sausages, and pizza) is an independent risk factor for gestational diabetes. Further research to confirm these findings in other populations and to decipher underlying molecular mechanisms is warranted. Nevertheless, our results emphasize the potential importance of considering pregravid dietary recommendations for the prevention of gestational diabetes. The information on the increased gestational risk associated with fast food intake might be disseminated to women of reproductive age.

## Supporting Information

Checklist S1 **STROBE Checklist.**
(DOC)
